# Regional Diets Targeting Gut Microbial Dynamics to Support Prolonged Healthspan

**DOI:** 10.3389/fmicb.2021.659465

**Published:** 2021-04-29

**Authors:** Dorrain Yanwen Low, Sophia Hejndorf, Rachel Thomas Tharmabalan, Sibrandes Poppema, Sven Pettersson

**Affiliations:** ^1^Lee Kong Chian School of Medicine, Nanyang Technological University, Singapore, Singapore; ^2^Department of Odontology, Karolinska Institutet, Solna, Sweden; ^3^School of Hospitality, Sunway University, Subang Jaya, Malaysia; ^4^School of Medical and Life Sciences, Sunway University, Subang Jaya, Malaysia; ^5^National Neuroscience Institute, Singapore, Singapore

**Keywords:** gut microbiome, gut microbiota, healthy ageing, prolonged healthspan, personalised diet, bioactive compounds, phytonutrients

## Abstract

In the last 150 years, we have seen a significant increase in average life expectancy, associated with a shift from infectious to non-communicable diseases. The rising incidence of these diseases, for which age is often the largest risk factor, highlights the need for contemporary societies to improve healthy ageing for their growing silver generations. As ageing is an inevitable, non-reversing and highly individualised process, we need to better understand how non-genetic factors like diet choices and commensal gut microbes can modulate the biology of ageing. In this review, we discuss how geographical and ethnic variations influence habitual dietary patterns, nutrient structure, and gut microbial profiles with potential impact on the human healthspan. Several gut microbial genera have been associated with healthy elderly populations but are highly variable across populations. It seems unlikely that a universal pro-longevity gut microbiome exists. Rather, the optimal microbiome appears to be conditional on the microbial functionality acting on regional- and ethnicity-specific trends driven by cultural food context. We also highlight dietary and microbial factors that have been observed to elicit individual and clustered biological responses. Finally, we identify next generation avenues to modify otherwise fixed host functions and the individual ageing trajectory by manipulating the malleable gut microbiome with regionally adapted, personalised food intervention regimens targeted at prolonging human healthspan.

## Ageing Is a Variable and Personalised Process

While ageing is an inherent genetically determined biological process, the chronological outcome of an individual’s lifespan is extraordinarily variable. Remarkably, so-called “blue zones” have been identified around the world where the proportion of centenarians (i.e., people who are 100 years old or more) is significantly higher than in neighbouring communities (Buettner and Skemp, 2016), but there are no validated biological explanations for these age-privileged cultural hotspots. At present, the global centenarian population is estimated to be between 500,000 and 600,000 (Robine and Cubaynes, 2017).

Ageing is associated with life-long accumulation of molecular damage that results in cellular deterioration and impairment in organ functionality and crosstalk, ultimately leading to collapse of body physiology and death (Melzer et al., 2020). The cellular and molecular damage that drive the ageing process may arise from genetic, environmental, and lifestyle factors. The heritability of lifespan is estimated in genome-wide association studies (GWAS) and twin studies to be ∼25% (Herskind et al., 1996), implying that up to 75% of our lifespan is determined by environmental and/or lifestyle factors.

**FIGURE 1:**
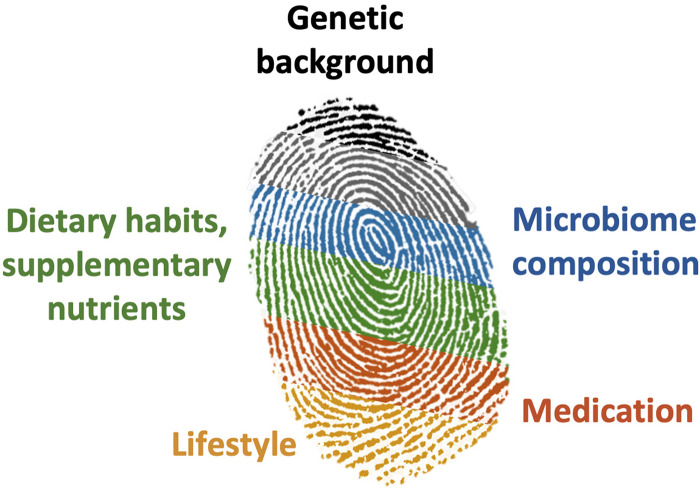
An individual’s healthspan is determined by inter-organ crosstalk of genetic, and modifiable environmental and lifestyle events that may be illustrated as a characteristic fingerprint, which influences body physiology, metabolism, and excretion.

However, a long chronological life may not be equivalent to a long healthspan (i.e., number of years spent in good health). While some estimates of genetic contribution to healthspan have been proposed (Ruby et al., 2018), we are in the early stages of understanding the factors that determine the onset and occurrence of age-related non-communicable diseases (e.g., cancers, neurodegeneration, osteoporosis, fractures, and cardiovascular diseases). Current estimates of heritability of non-communicable diseases average 40% but vary greatly from <10% in Parkinson’s disease to >60% in osteoporosis (Steves et al., 2012). This suggests that modifiable environmental and lifestyle factors (e.g., diet, gut microbiota; Figure 1) represent an avenue to prolong healthspan.

**FIGURE 2:**
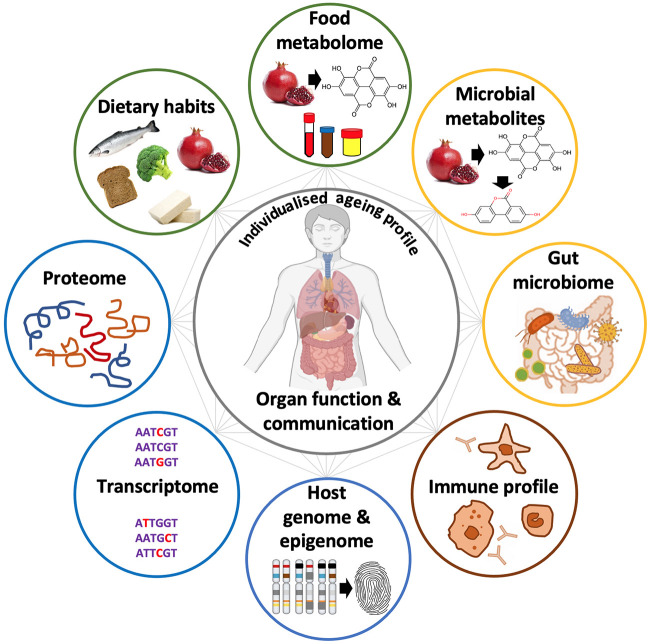
Life-long accumulation of damage to body functions and organ systems leads to a discrepancy between chronological and biological ageing. Further, these individual nodes and inter-organ crosstalk may contribute to variability of an individual’s ageing process.

In recent decades, we have seen remarkable progress in understanding how molecular pathways are linked to the ageing process, leading to the conceptualisation of nine molecular “Hallmarks of Ageing” (López-Otín et al., 2013), and spurring research into modifiable or druggable molecular targets within each hallmark (Campisi et al., 2019). Despite sharing common hallmarks, the ageing process is still highly individualised as evident by the increased variability in organ function and inter-organ crosstalk (Steves et al., 2012). For example, Ahadi et al. (2020) characterised the variability of ageing with 184 individual molecular markers, which can be grouped into four partially overlapping domains of ageing or ageotypes: immunological, metabolic, hepatic, and renal. They observed that individuals tended to age asymmetrically, displaying different rates of ageing between the domains (Ahadi et al., 2020). Hence, to achieve a comprehensive and clinically applicable understanding of ageing, insights into common molecular principles of ageing must be accompanied by a decipherment of variability and individuality of the ageing process as well as underlying and modifiable factors behind this variability (Figure 2). The imminent need to address how environmental and lifestyle factors drive the individuality of the ageing process is therefore highly warranted and certainly within the vision of the twenty-first century of precision medicine.

## Dynamic Gut Microbiome as an Accessible Target for Healthspan Interventions

**FIGURE 3:**
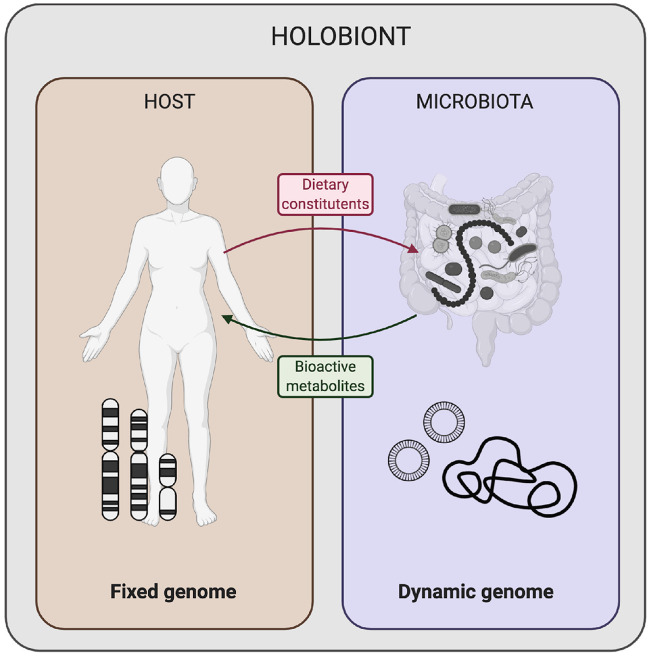
The holobiont genome comprise of the human host genome (fixed at conception with limited accumulation of somatic mutations throughout life) and dynamic microbiome (amenable to change following dietary variations). Dietary intake and habits of the human host can determine the genomic configuration and transcriptional activity of the commensal gut microbiota that break down ingested foods to produce bioactive metabolites with a wide range of effects on the human host. Illustration was created with BioRender.com.

Increasingly, gut microbes and their plethora of genes and secreted molecules are being recognised as an integral part of the holobiont (Figure 3), where the human host and prokaryotic microbial counterparts coexist to mutually benefit and function as a meta-organism (Byndloss and Bäumler, 2018). While the human genome is fixed at birth, the gut microbiome is dynamic, characterised by a rapid cellular turnover (average of 5 days, in the human host) (Sender and Milo, 2021) and can be considered superior in its plasticity to transcriptionally respond to dietary interventions as compared to host eukaryotic cells (Figure 3). This highlights the potential to target the gut microbiota to alleviate, for example, accelerated ageing by dietary interventions. The observation that gut microbes display considerable changes in composition (and by extension, their functions as a community) in many age-associated non-communicable diseases (Byndloss and Bäumler, 2018) allows for prospective dietary interventions to be applied in situations where impairments in microbe-microbe functions and/or dysfunctional microbe-host interactions are driving the disease. It is tempting to speculate that the spectrum of symptoms associated with accelerated ageing may in part be reversed or mitigated due to the dynamic nature of the gut microbiome.

Accumulating evidence points to diet as a central determinant of gut microbial composition. A study into South East Asian populations of various ethnic origins showed that diet overruled ethnicity, lifestyle, and environmental factors in determining the gut microbial configuration (Khine et al., 2019), confirming an Israeli cohort study, which found no significant association between genetic ancestry and microbial composition (Rothschild et al., 2018). In contrast, the Dutch HELIUS study identified ethnicity as a strong determinant of microbial composition [main operational taxonomic units (OTU) characterised as *Prevotella* in Moroccans, Turks, and Ghanaians, *Bacteroides* in African Surinamese and South-Asian Surinamese, and *Clostridales* in the Dutch] (Deschasaux et al., 2018). However, the same study also showed that there was a strong correlation between ethnicity and dietary pattern, suggesting that diet might account for inter-ethnic differences (Deschasaux et al., 2018). Additionally, ethnicity comprises other aspects such as cultural habits, socioeconomic status, health care, antibiotics use, and early-life environment, which may all contribute to shape the gut microbiome in a specific fashion by determining nutrient availability and xenobiotic exposure. Furthermore, differences in early-life colonisation trajectories by maternal microbes may shape organ-to-organ communication, immune system development, maturation of epithelial linings, and tuning of brain development and function.

## The Diet-Microbiome Axis Is Implicated in Healthy Ageing

Dietary pattern and gut microbial composition correlate with longevity and markers of healthy ageing. Simultaneously, the dynamic interaction between diet and microbes is interwoven: diet regulates the phylogenetic structure and biological activity of the gut microbes, whereas the microbes, in turn, regulate the presence and/or bioactivity of food molecules through their metabolism and biotransformation (Rowland et al., 2018). Hence, understanding the relationship between diet, gut microbes, diet-gut microbe interactions, and host organ function is of utmost importance if we aim to intervene in the individualised ageing process to improve health and healthspan in the ageing population through personalised nutritional interventions.

### Gut Microbiome and Longevity

**FIGURE 4:**
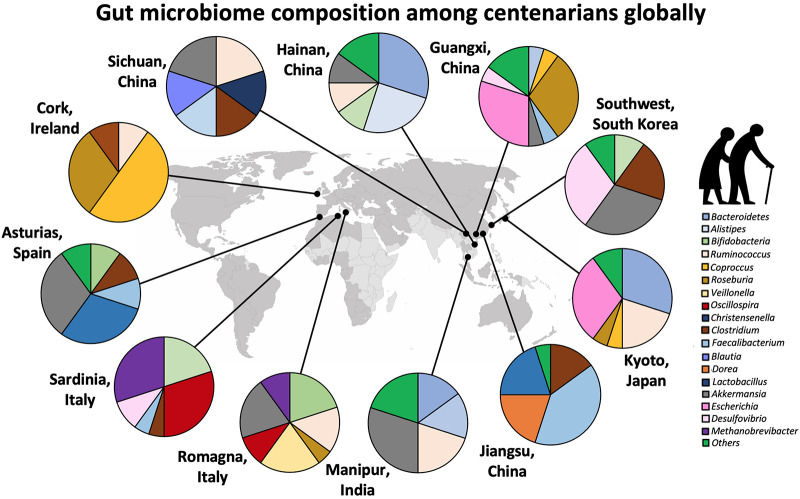
Centenarians harbour distinct gut microbial species and community structures unique to the region, suggesting that microbial functional and biochemical properties are pivotal to healthy ageing, rather than the phylogenetic composition. Data adapted from Claesson et al. (2012), Fang et al. (2015), Biagi et al. (2016), Kong et al. (2016), Bian et al. (2017); Bong-Soo et al. (2019), Naito et al. (2019), Salazar et al. (2019), Tuikhar et al. (2019), Wu et al. (2019), and Luan et al. (2020).

Centenarians, who have escaped or survived lethal diseases earlier in life, may be considered a spontaneous model of healthy ageing. The gut microbial composition of centenarians has consistently been reported to differ in phylogenetic composition from that of younger people. Interestingly, within centenarian populations, species have been reported to display regional characteristics, further supporting that environmental and/or lifestyle factors including the diet, shape microbial composition (Figure 4). For example, in an Italian cohort, the centenarian microbiome was found to be dominated by the same two microbial families as in the other age groups (<75 years old) of the population, namely *Veillonellaceae* and *Ruminococcaceae* (*Firmicutes* phylum), but was specifically enriched in the genera *Akkermansia*, *Bifidobacterium*, and *Christensenella* (Biagi et al., 2016). In contrast, the Chinese Hainan Centenarian Cohort was dominated by *Bacteroides* (*Bacteroidetes* phylum) and *Escherichia* (*Proteobacteria* phylum) (Luan et al., 2020). Long-term elderly care residents in the Irish ELDERMET Cohort also had a gut microbiome dominated by *Bacteroidetes* (Claesson et al., 2012). Importantly, although the aggregate faecal microbiome in ELDERMET was dominated by *Bacteroidetes*, the residents showed extraordinary inter-individual variation with 3–92% *Bacteroidetes* and 7–94% *Firmicutes*, hinting at a long-term effect of their dietary habits (Claesson et al., 2012).

The results of the Italian study are also in contrast to those of another Chinese Centenarian Cohort from the Guangxi region, who harboured significantly higher abundance of the genera *Escherichia* and *Roseburia*, and reduced abundance of *Akkermansia*, *Lactobacillus*, *Faecalibacterium*, *Parabacteroides*, *Butyricimonas*, *Coprococcus*, *Megamonas*, *Mitsuokella*, and *Sutterella* (Fang et al., 2015). A Korean centenarian study found trends similar to both Italian and Guangxi Chinese centenarians, with higher abundance of *Akkermansia and Christensenella*, and *Escherichia*, respectively. They also displayed increased abundance of *Clostridium* and *Collinsella*, and reduced abundance of *Faecalibacterium* and *Prevotella* compared to the general population (Bong-Soo et al., 2019).

At present, we do not have a good understanding to explain these geographical variations in the centenarian gut microbial composition or to unequivocally answer if there are certain microbial species globally associated with longevity. In the reviewed studies (Supplementary Table 1), some microbial genera associated with healthy elderly populations include *Roseburia*, *Escherichia*, *Akkermansia*, *Christensenella*, *Bifidobacterium*, and *Clostridium*, but they are all highly variable across populations. Based on these cross-sectional observations, it seems unlikely that a universal pro-longevity gut microbiome exists. Rather, the optimal microbiome for healthspan appears to be conditional on the microbial functionality acting on regional- and ethnicity-specific trends driven by cultural food context (Supplementary Table 1). Furthermore, because lifespan is influenced by exposures throughout life and the microbiome tends to change over time, the gut microbiome snapshot of centenarians might not capture all the microbial factors contributing to their long life leading up to the time of measurement. For example, Wilmanski et al. (2020) recently reported that the “uniqueness” of the gut microbiome, which correlates with health parameters, starts significantly increasing from the age window of 40–50 years and with each subsequent decade. Asserting that the centenarian microbiome represents a pro-longevity microbial configuration would require individually tracked longitudinal studies of pre-centenarian cohorts.

Additionally, the phylogenetic composition of the gut microbiome might not accurately represent the functional output as species across genera are often able to perform similar metabolic processes. It is of interest for future studies to complement phylogenetic analysis with metabolomics and proteomics of the host blood and faecal material. For example, the abundance of microbial tryptophan synthase (TrpB) and tryptophanase (TnaA) responsible for synthesising tryptophan and indole, respectively, was reduced in a Spanish cohort of elderly (65–85 years old) compared to infants (2–5 years old) and young adults (24–45 years old) (Ruiz-Ruiz et al., 2020). Homologues of TnaA were previously identified in >85 species in a variety of genera including *Escherichia*, *Shigella*, *Porphyromonas*, *Clostridium*, *Enterobacter*, etc. (Lee and Lee, 2009).

### Diet and Longevity

In the Western world, the Mediterranean Diet (MedDiet) is widely regarded as a “healthy diet” and adherence to this diet or diets with similar macronutrients content has been associated with longevity and reduced risks of age-related non-communicable diseases in culturally diverse populations (Roman et al., 2008). There is no consensus on the exact MedDiet structure, but it is characterised by high consumption of plant-based foods rich in whole grains, vegetables, fruits, nuts, legumes, and olive oil, followed by moderate consumption of fish, dairy products, and low consumption of red meat.

However, not all longevity regions exhibit adherence to the MedDiet structure. For instance, centenarians from Hainan, a region recognised by the International Expert Committee of Population Aging and Longevity as a World Longevity Island for its highest percentage of centenarians (18.75/100,000) in China, scored only 7.7 (±1.9) out of 18 on the MEDI-LITE adherence score, significantly lower than their Italian counterparts (Luan et al., 2020). This suggests that although the MedDiet pattern seems beneficial across genetic groups and cultures, some regions may benefit from developing dietary guidelines better suited to their geographical and cultural climates. Additional studies evaluating the effects of nutritional intervention on diseases, ageing, and longevity must consider geographical variation.

## Regional Dietary Patterns and Associated Gut Microbiome Trends

Dietary patterns, stemming from variations in culture, beliefs, and availability of food across seasons, are evidently different regionally and an important determinant driving gut microbial diversity and richness. Generally, shifting from a low-fat, high-fibre (“healthy”) diet to a high-fat, high-sugar, high-protein, low-fibre (“unhealthy”) diet leads to decreased α-diversity (i.e., intra-individual microbial richness), increased β-diversity (i.e., inter-individual microbial diversity), and decreased abundance of species (e.g., *Prevotella* and *Treponema*) (De Filippo et al., 2010). Furthermore, food additives commonly used in ultra-processed foods (e.g., emulsifiers, artificial sweeteners, salt) and Maillard reaction products (e.g., pyrazines and furans that are formed during thermal processing) were recently demonstrated to decrease α-diversity in the *Milieu Intérieur* study, while increasing the *Firmicutes:Bacteroidetes* (*F:B*) ratio (Partula et al., 2019), which has been previously associated with obesity and cardiovascular diseases.

In Asia, populations typically consume a high-starch diet based on rice or noodle, different to the Western diet rich in animal meat and processed foods or to the Nordic diet rich in whole grain cereals, fatty fish, berries and root vegetables. A carbohydrate-rich diet was shown to select for the beneficial *Bifidobacterium* genus in Indian and Chinese living in Singapore (Jain et al., 2018), a small island state (5.8 million population, land area 726 km^2^) in the Straits of Malacca containing diverse ethnic populations. High *Bifidobacterium* abundance was similarly observed in Asian children living in China, Taiwan, Japan, Indonesia and Thailand (Nakayama et al., 2015), and Japanese adults (Nishijima et al., 2016) as expected based on this genus’ higher expression of glycoside hydrolases for degrading starch relative to other intestinal microbes. Other genera, significantly more represented in Singaporean Indians compared to Singaporean Chinese were *Bacteroidetes* (4-times higher), *Prevotella* (21-times higher), *Megasphaera*, *Catenibacterium*, *Lactobacillus*, *Mitsuokella*, *Carnobacterium*, and *Lachnospira* (Fukkoshi et al., 2015), pointing to a gut microbe determining role of dietary components characteristic of Indian plant-based diet- heavily spiced and curried foods, ghee, lentils and coconut milk. For example, basmati rice, commonly used in Indian cuisine, has a lower glycaemic index and higher amylose-amylopectin ratio compared to other medium- or long-grain rice (Kaur et al., 2014). In contrast, the Singaporean Chinese diet is characterised by noodles, white bread, animal protein and fat, seafood, soy-based products, and heavily sweetened beverages.

Lee et al. (2017) found that globally, populations with *Prevotella*-containing gut microbiota, e.g., Indonesian, Thai, Korean, and African, share a similarity in reduced meat consumption compared to populations harbouring a higher proportion of *Bacteroides* and *Bifidobacterium*. Likewise, significant associations were detected between vegetable-based diets and increased abundance of *Prevotella* and fibre-degrading *Firmicutes* in an Italian cohort as well as increased levels of faecal short chain fatty acids (SCFA) (De Filippis et al., 2016). Interestingly, a recent paper showed that the gut microbiome of vegetarians and vegans had developed selective responses to plant-based diets rich in slowly digestible and complex carbohydrates, including increased cell motility (e.g., flagellin) to physically access nutrients, increased catalytic activities for carbohydrate and food proteins as well as the synthesis/release of bioactive compounds (De Angelis et al., 2020). In a separate study, the gut microbial composition of people with a high MedDiet adherence was found to be phylogenetically diverse, including broadly anaerobic fermenters, and more niche- and subject-specific biochemical specialists as well as major dietary fibre metabolisers (e.g., *Faecalibacterium prausnitzii*, *Eubacterium eligens*, and *Bacteroides cellulosilyticus*) (Wang et al., 2020). Their gut microbiomes were enriched for bacterial metabolism of plant-derived polysaccharide degradation, SCFA production, and secondary bile acid biosynthesis (Wang et al., 2020).

The gut microbiota of Southern Chinese population in two urbanised Malaysian (32 million population, land area 330,000 km^2^) regions, Penang City (west coast) and Kelantan City (east coast), was abundant in *Bifidobacterium* and *Collinsella*, both positively correlating with refined sugar-enriched foods (Khine et al., 2019). Additionally, *Collinsella* was positively correlated with fruits and curried foods but negatively correlated with Southeast Asian vegetables, while *Bacteroides*, *Fecalibacterium and Bifidobacterium* were negatively correlated with caffeinated drinks, curried and oily foods (Khine et al., 2019). The same study did not find significant differences in microbial composition between ethnicities living in the same location. This is not surprising as Malaysian cities are populated by Malay and Chinese ethnicities, and most residents are thus exposed to and share a fusion of Malay and Chinese food cultures, e.g., Peranakan (or Nyonya) food- a blend of Chinese ingredients with distinct Malay spices or cooking methods. These dishes are pungent and often use coconut milk and galangal. Peranakans have settled mainly in Penang or Malacca with slight variations to their cuisine (Chung, 2019). Nevertheless, preferred foods are a personal choice and may explain the segregation of microbial composition between clusters within a population or city.

Some Malaysian regions are populated by forest-dwelling hunter-gatherer tribes, also known as the Orang Asli. In a study comparing the effects of ethnicity and socioeconomic status on gut microbiota profiles, pre-adolescents of Orang Asli were observed to possess richer microbial diversity compared to urbanised Malay and Chinese cohorts (Chong et al., 2015). *Ruminococcaceae* and a *Spirochaetes*-related 16S rRNA gene signature were enriched in the former gut microbial profiles, which have been previously associated with the breakdown of fibre-rich food (Chong et al., 2015). The *F:B* ratio in Orang Asli was 6 times lower than other Malaysian and Myanmarese populations (Chua et al., 2019), and can be linked to their primarily plant-based diet, reflecting their horticultural and hunting lifestyle. It is worth noting that the oral microbial composition and functional profiles between Orang Asli sub-groups differ depending on location and gender (Yeo et al., 2019). Extensive gender-specific variation in their gut microbial composition can be observed, likely due to food taboos, where women and children are restricted against the intake of certain foods (e.g., game animals, jackfruit, coconut, and “ikan kelah”) while men are allowed to consume a wider variety of wild and game animals.

Another example of regional dietary influence is the higher prevalence of porphyranase- and agarase-encoding genes in the gut microbiome of Japanese and Chinese populations than in North American and European populations (Hehemann et al., 2010; Pudlo et al., 2020). These genes encode proteins that break down complex glycosidic linkages of porphyrapolysaccharides. They were originally reported to have transferred from *Zobellia galactanivorans* derived from Nori red algae (a significant portion of the Japanese diet) to *Bacteroides plebeius* while passing through the gastrointestinal tract (Hehemann et al., 2010). *B. plebeius* was found amongst the top five species in the Singapore elderly BAMMBE cohort; its presence did not correlate with the low consumption of sushi in this cohort but likely reflects the higher level functionality of this genera of bacteria, which is responsible for producing Carbohydrate Active Enzymes (CAZymes) involved in synthesis, recognition, or metabolism of complex carbohydrates (i.e., oligosaccharides, polysaccharides, glycoconjugates). Interestingly, high levels of genes encoding for plant-degrading enzymes were found in the modern Hadza hunter-gatherer tribes of Tanzania, whose diet consists mainly of plant-based fibre-rich and unprocessed foods, whereas genes encoding enzymes targeted towards animal and mucin degradation were enriched in Americans (De Filippo et al., 2010; Fragiadakis et al., 2019), aligning with industrialisation and urbanisation. Alike the Orang Asli, *Spirochaetaceae* was similarly found in hunter-gatherer and agrarian populations, as was *Prevotellaceae*, *Succinovibrionaceae*, and *Paraprevotellaceae* (Fragiadakis et al., 2019).

Enriched levels of *B. plebeius* were also detected in a Korean sub-population (Tuikhar et al., 2019), where Nori is frequently consumed. A similarity between the diets of Korean (52 million population, land area 100,200 km^2^) and Japanese (127 million population, land area 378,000 km^2^) is the heavy consumption of fermented foods such as fermented vegetables (e.g., kimchi, natto, and tsukemono), soybean paste, fish products, red pepper paste, medicinal herbs, and sesame or perilla oil (Kim et al., 2016), originally developed to cover for shortfalls in food during winter. The intakes of fermented legumes, vegetables, and potatoes were found to be positively associated with higher α-diversity and *F:B* ratio in the Korean NAS-IARC cohort (Noh et al., 2021). A metagenomic analysis of kimchi revealed dominating members of *Leuconostoc*, *Lactobacillus*, and *Weissella* (Jung et al., 2011), with the latter recently isolated and identified in traditional Indian fermented foods (Månberger et al., 2020). *Leuconostoc* species were also previously found in participants of the Dutch Lifelines (DEEP) cohort who specifically consumed a fermented milk product, buttermilk (Zhernakova et al., 2016). However, it is not known how the proportion of these genera are distributed in the gut microbiota of other regional populations. Fermented foods (i.e., yoghurt) are a known source of probiotics, which are thought to bring about gastrointestinal health benefits such as increased microbial diversity and enriched resident microbiota when consumed in adequate amounts (Marco et al., 2017). Kefir, a fermented milk beverage made from colonies of lactose-fermenting yeast, and lactic- and acetic acid-producing bacteria, is gaining popularity in recent years, and has been suggested to reduce lactose malabsorption and promote *Helicobacter pylori* eradication (Dimidi et al., 2019). However, robust clinical evidence from randomised controlled human trials confirming the effects of various fermented foods on gastrointestinal health are limited.

These studies, collectively, indicate local and global variations in the human gut microbiome, which are attributed to dietary cultures and choices. A common nutritional feature of healthy or longevity diets seems to be the frequent consumption of a minimally processed plant-based diet rich in complex carbohydrates, fruits, vegetables, soy-bean based foods, nuts, and seafood, which emphasises a “healthy fat” profile (i.e., higher in unsaturated fats and omega-3, and lower in saturated fat). The healthy fat being a likely mechanism for reducing inflammation, optimising cholesterol and other risk factors (Nishijima et al., 2016), and when combined with the lower caloric density of plant-rich diets and concomitant high intake of bioactive phytonutrients, jointly reduce risk for chronic age-related diseases and promote healthy ageing and longevity.

## Mapping the Bi-Directional Interactions Between Dietary Components and Gut Microbiota to Understand Variability in Inter-Individual Response

Studies of how diet affects healthspan are commonly limited to macronutrients (e.g., sugar, fat, vitamins) and structural components (e.g., dietary fibres). However, these nutritional components represent a small fraction of >26,000 phytonutrients described in foods^1^ ^,2^ such as polyphenols, terpenoids, alkaloids, and other plant secondary metabolites. Some of these compounds have been shown to nourish the gut microbiome, which in turn, metabolises these precursors into smaller molecular weight compounds, of which many serve a regulatory role. For instance, olive oil, a well-known ingredient in the MedDiet, has more than 200 unique compounds listed in specialised food metabolome databases (Kabaran, 2018), but only 60 and 8 common nutritional components in the US FoodData and Singapore Health Promotion Board nutrient composition databases, respectively. Barabasi adeptly described this untracked diversity of compounds in foods as the “dark matter of nutrition,” which remains largely invisible to epidemiological or hypothesis-driven nutritional studies (Barabási et al., 2020). Considering the chemical diversity of diets in different regions, it is necessary to further our understanding of the full chemical composition of specific foods and complex diets, to be able to map how our highly individualised gut microbiome responds to varying combinations of phytonutrients in geographically or culturally restricted fashions, and how to best employ the gut microbiome for personalised next-generation dietary interventions. In Supplementary Table 2, we highlight regional examples of known phytonutrients and functional foods, and their gut microbiome-associated trends that elicit individual and clustered biological responses that could be employed to improve health in an ageing population.

## Next Generation Dietary Interventions and Prolonged Healthspan: Challenges and Opportunities

An individual’s genome may be fixed at conception, but its gut microbiome remains extraordinarily malleable throughout life with a fluctuating genetic composition that is able to respond to both host and dietary cues. By manipulating and utilising the diverse genetic pool of the gut microbiome with targeted dietary interventions, there is an untapped potential to complement and modify otherwise fixed host functions. Most dietary recommendations for the general population represent a “one-size-fits-all approach,” which does not ensure that everyone has adequate exposure to health-promoting constituents of foods. For example, dieticians have been using standardised glycaemic indices of food items to guide dietary recommendations in the last decade, but Zeevi et al. (2015) recently showed that glycaemic response to identical foods is in fact highly variable between people and largely dependent on their gut microbiome.

**FIGURE 5:**
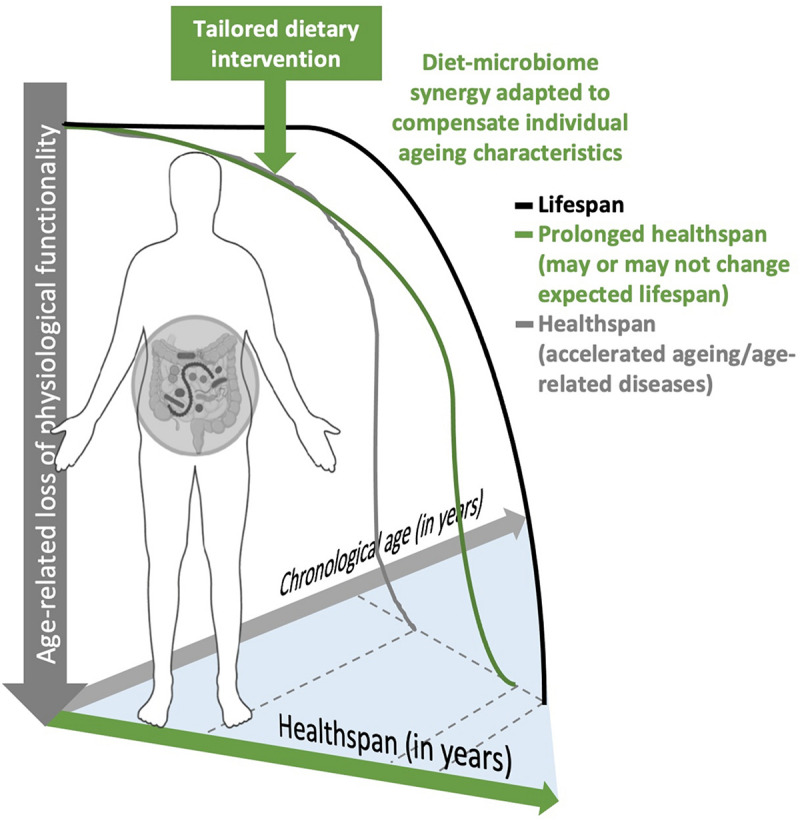
An illustration of how physiology (individualised ageing profile) and host-microbiome synergy may characterise an individual approaching their twilight. Introducing regionally adapted, personalised dietary intervention to select for or nurture the malleable gut microbiome may alter the ageing trajectory to compensate for age-related loss of physiological functionality.

From experience, we have learned to tailor-make food products to certain age and/or vulnerable groups. For example, nutrient-rich milk formula supplemented with essential fatty acids and nucleotides have been designed to meet nutritional demands for the growth spurt of infants, and a selection of ready-to-eat weaning and solid products (e.g., soft vegetable or fruit puree) have been developed for toddlers to transition into whole diets. At the other bookend of life, liquid or pureed food products spiked with nutrients and dietary fibres have been developed to cater to the elderly’s reduced capacity for nutrient absorption, decreased taste and smell perception, as well as dental problems resulting in chewing difficulties. Crucially, these existing food products for the elderly are limited in scope to alleviate nutritional shortcomings. A large potential exists in shifting the perspective towards developing a next generation of dietary intervention that shapes, nurtures, and utilises the gut microbiome to complement naturally deteriorating host functions in ageing (Figure 5).

Leveraging on the structural composition of food matrices (e.g., whole, intact foods, slowly digestible complex plant carbohydrates, and less processed/liquified foods) is therefore a promising approach to promote longer retention time in the large intestine (Low et al., 2021) to stimulate microbial diversity, richness and a shift in microbial metabolism from protein catabolism towards carbohydrate fermentation (Grant et al., 2019). To develop guidelines for personalised (and next generation) dietary interventions, there is an urgent need to allocate sufficient resources for basic and translational research that aim to unravel the mechanisms of how physical factors of food matrices alter gut microbial composition and function.

Developing a next generation of dietary interventions for prolonged healthspan requires documentation of individual variation at a systems biology level. A compilation of clinical parameters, socio-economic status, and multi-omics analyses measuring host, diet, and microbial-derived metabolites and microbiome configuration will allow a more accurate determination of an elderly individual’s body physiology relevant to the chronological age and estimation of healthspan trajectory. Recent developments in food metabolome databases and innovative metabotyping technologies present opportunities for improving profiling of habitual dietary patterns. A deep characterisation of predictors of individuals’ varied response to dietary components can inform well-defined targets or stratified population clusters and guide food companies for the future design of next generation food products. It may be a central component of personalised dietary intervention studies to generate and meaningfully analyse vast amounts of systems level data. This can be challenging since enormous effort, sufficiently large well-phenotyped cohorts, and big data software architecture are required for feeding into machine learning algorithms to be able to recognise patterns that characterise an individual’s health and vulnerability (e.g., absence of certain gut microbial communities or lack of consumption of certain food components), and to predict their response to the corresponding dietary interventions. Moreover, it is essential to consider the effect of regional differences in food cultures and lifestyle on baseline microbial and physiological features that will describe the response to new dietary interventions.

The ageing population presents a suitable target for implementing dietary interventions as they have been demonstrated to be receptive towards changing their dietary habits to achieve specific health outcomes (Berendsen et al., 2018). Furthermore, they represent a population of high risk of disease mortality as critically illustrated by the COVID-19 pandemic, which may be alleviated by administering tailored dietary interventions to ensure a strong and vigilant immune system ready to defend the human host under conditions of metabolic homeostasis and optimal inter-organ crosstalk, thus making humans more resilient to emerging stressors.

In summary, we must strive to better define individuals across an otherwise invariant chronological age range, to identify those at greater risk of accelerated ageing for healthspan intervention. Modifiable environmental and lifestyle factors such as our diet and gut microbiome represent accessible targets for prolonging healthspan, where we may introduce personalised dietary interventions to select for or to nurture the highly malleable gut microbiome to alter the ageing trajectory. The design of future diet intervention strategies should consider the chemical diversity of foods and inter-individual variability in biological responses with an emphasis on regional- and cultural-specific context.

## Author Contributions

DL, SH, and SvP contributed to conception of the review. DL and SH drafted the manuscript. All authors reviewed and approved the manuscript.

## Conflict of Interest

The authors declare that the research was conducted in the absence of any commercial or financial relationships that could be construed as a potential conflict of interest.

## Footnotes

1 https://foodb.ca

2 http://phytohub.eu
