# Supplementary material for: Regional Diets Targeting Gut Microbial Dynamics to Support Prolonged Healthspan
Source: Front Microbiol. 2021 Apr 29;12:659465. doi: 10.3389/fmicb.2021.659465 (PMC8116520; doi:10.3389/fmicb.2021.659465)
Supplement: Supplementary file 1 [file Table_1.pdf]

Supplementary Table 1. Review of publications reporting regional and dietary associations with the gut/faecal microbiome in elderly and centenarian populations.

Geography	Cohort details	Findings	Diet characteristics or identified clusters	References
Cork city and county region, Ireland	ELDERMET Cohort, 178 Caucasians (64-102 years old)	• Long-term residential care residents had ↑ *Bacteroidetes* (phylum level); *Parabacteroides, Eubacterium, Anaerotruncus, Lactonifactor and Coprobacillus* (genus level).<br>• Community-dwelling residents had ↑ *Firmicutes* (phylum level)*; Coprococcus, and Roseburia* (genus level).<br>• *Clostridiales was* associated with frailty and malnutrition.<br>• *Bacteroides* was associated with diets rich in animal produces, and low in fruits and vegetables.<br>• Ageing was accompanied by lower consumption and variety of fiber intake → reduced microbiota diversity → detrimental to gut health.	• Dietary patterns in community location correlate with microbial composition differences.<br>• Dietary groups (DG) can be clustered into:<br>- DG1: complex carbohydrates (CHO), fruits and vegetables daily, protein-rich white meat/fish/eggs 5 times/week, dairy 3 times/week.<br>- DG2: complex and simple CHO, red meat/fish/eggs daily, fruits and vegetables 2-3 times/week, dairy produce once/week.<br>- DG3: least variety, simple CHO.	(Claesson et al., 2012; Wu et al., 2019)
Emilia Romagna, Italy	Semi-supercentenarians (105-109 years old), centenarians (99-104 years old), 13 elderly (65-75 years old), 11 adults (22-48 years old)	• Ageing was associated with:<br>- ↑ abundance of *Christensenellaceae* (family level); *Akkermansia, Methanobrevibacter, Bifidobacterium* and *Oscillospira* (genus level).<br>- ↓ *Ruminocoacea,* Coprococcus and *Roseburia* (genus level); *Bifidobacterium adolescentis*, *Bifido- bacterium longum*, *Bacteroides uniformis*, *Faecalibacterium prausnitzii*, *Ruminococcus bromii*, *Subdoligranulum* sp., *Anaerostipes hadrus*, *Blautia obeum*, *Ruminococcus torques*, *Coprococcus catus*, *Coprococcus comes*, *Dorea longicatena,* and *Roseburia sp* (species level).<br>• A progressive age-related ↑ in number of reads for genes devoted to xenobiotic biodegradation and metabolism, and ↓ in CAZymes genes involved in CHO metabolism.<br>• Age-related differences in lipid metabolism (α-linoleic acid, glycerolipid), lipopolysaccharide biosynthesis and certain amino acid metabolism (tryptophan, tyrosine, glycine, serine and threonine).	• Diet is characterised by pasta, bread, meat, salami, fish, wild fruits and vegetables, olives, cheese and generous seasonings.	(Biagi et al., 2016; Rampelli et al., 2020; Zicasso)
Sardinia, Italy	19 centenarians (99-107 years old), 23 elderly (68-88 years old), 17 adults (21-33 years old)	• ↑ diversity of core species and microbial genes in centenarians than in elderly and adults.<br>• ↑ *Pyramidobacter, Desulfovibrio* (genus level); *Methanobrevibacter smithii*, *Bifidobacterium adolescentis* (species level) and ↓ *Ruminococcus, Corprococcus, Dorea*	• Mediterranean-style plant-based diet comprising of fruits, vegetables, legumes, nuts, olive oil, whole wheat bread, moderate consumption of red wine (e.g., cannonau), goat's milk, yoghurt (e.g., goiddu) and low consumption of animal-based foods.	(Barbagallo et al., 2002; Wu et al., 2019)

		(genus level); *Faecalibacterium prausnitzii, Eubacterium rectale* (species level) in centenarians.<br>• Gut microbiota was correlated with host functional independence in centenarians:<br>- High capacity for central metabolism (glycolysis and fermentation to SCFAs) but low in gene-encoding enzymes involved in CHO degradation.		
Asturias region, Spain	49 adults (<50 years old), 58 adults (50-65 years old), 19 elderly (66-80 years old), 27 elderly (>80 years old)	• ↑ *Akkermansia* and *Lactobacillus* (genus level) in >80 years old.<br>• ↓ in *Bifidobacterium*, *Faecalibacterium*, *Bacteroides* and *Clostridium* cluster XIVa (genus level) with age.<br>• Ageing was associated with ↓ faecal SCFA.<br>• Levels of *Bacteroides* ↓ correlated with intake of polyunsaturated fatty acids but ↑ with vitamins B_6 and C.<br>• Levels of *Bacteroides* and *Clostridium* cluster XIVa ↑ correlated with intake of polyphenols.<br>• Levels of *Lactobacillus* ↓ correlated with intake of vitamins A and D.	• Asturian diet is characterised by fresh fish, cheese, beans, cider and red wine.<br>• Variable energy intake observed with age:<br>- <50 years old: 19 kcal/day<br>- 50-65 years old: 2033 kcal/day<br>- 66-80 years old: 1624 kcal/day<br>- >80 years old: 1728 kcal/day<br>• Elderly >80 years old have ↑ intake of saturated and polyunsaturated fatty acids, but ↓ intakes of carotenes, folic acid, polyphenols and vitamins A, C, D and B_6.	(Salazar et al., 2019)
Sichuan, China	168 Chinese, elderly (>90 years old) compared to elderly and adults	• In long-living individuals, ↑ community richness (Chao index) and diversity (Shannon index), as well as ↑ abundance of and Erysipelotrechaceae (family level); *Akkermansia, Ruminococcus, Christensenella, Clostriduim XIVa, Blautia members, Faecalibacterium* (genus level).	• Common foods consumed include wild game, seafood, fresh vegetables, bamboo shoots, mushrooms and fungus.<br>• Diverse variety of fine-textured fermented and cultivated condiments: Zigong well salt, Neijiang white sugar, Langzhong Baoning vinegar, Deyang soy sauce, Pixian bean paste, Maowen and Chengdu pepper, Xufu sprouts and Nanchong Dongcai.	(Kong et al., 2016; Zhang and Ma, 2020)
Zhenjiang, Suzhou and Nantong cities, Jiangsu Province, China	>1000 healthy Chinese (3-100 years old)	• In older individuals, ↑ in *Dorea, Clostridium insertae sedis and sensu strictu 1* and *Marvinbryantia* (genus level) and to a lesser extent, members of *Prevotella* genus.<br>• Older individuals microbiome revealed little difference to younger individuals, likely as they were exceptionally healthy, lived in a single region and ate a very similar diet.<br>• Clear regional difference, where members of *Faecalibacterium* genus form core microbiota in this population.	• Diet is characterised by varied consumption of seafood (e.g., catfish, crab, sturgeon, long-tailed anchovy).<br>• Common specialty dishes in Jiangsu province include watermelon chicken, sweet and sour mandarin fish.<br>• Predominant vegetables include Xianghu water shield, Huaian cattail, Baoying lotus, Chinese chestnut, gorgon fruit, *Zizania* aquatic, winter bamboo shoots and water chestnut.	(Bian et al., 2017; Zhang and Ma, 2020)
Chengmai, Danzhou and Lingao districts, Hainan, China	China Hainan Centenarian Cohort, 75 centenarians (96-110 years old)	• Significant ↓ in *Akkermansia muciniphila*, *Alistipes finegoldii*, *Alistipes shahii*, *Bacteroides faecis*, *Bacteroides intestinalis*, *Butyrivibrio crossotus*, *Bacteroides stercoris*, and *Prevotella stercorea* (species level).<br>• ↑ in *Bifidobacterium longum* and *Ruminococcus bromii* (species level) before death.	• Minimally-processed diet rich in fruits, wild vegetables, grain-based products (noodles, congee, bun) and meat (mainly pork) with cooking methods being mostly boiled or mild seasoning.<br>• Proportion of fats in dietary structure is slightly higher (33%) than MedDiet.	(Luan et al., 2020)

			• Averaged total food intake = 1,040 kcal/day.<br>• CHO contribute ~50% of total energy intake, lower than that of Italians and Asian immigrants in North Italy.	
Bama county and Nanning city, Guangxi, China	Centenarians (100-108 years old), elderly (85-99 years old) in Bama county, elderly (80-92 years old) in Nanning city	• ↑ abundance in *Roseburia* and *Escherichia* (genus level),<br>• ↓ in *Lactobacillus, Faecalibacterium, Parabacteroides, Butyricimonas, Coprococcus, Megamonas, Mitsuokella, Sutterella* and *Akkermansia* (genus level) in these centenarians.<br>• Diet-related OTUs were classified as *Bacteroidales* (order level) and *Lachnospiraceae* (family level) (↓ in high-fiber diet) and *Ruminococcaceae* (family level) (↑ in high-fiber diet).	• Bama diets are characterised as low-fat, low-calorie, high in vitamins and fibre (hemp, corn, brown rice and millet).<br>• Plant-based diet including pumpkin, tomatoes, amaranth leaves, peppers, sweet potatoes and their leaves, pak choi, mushrooms, bamboo shoots, peppers, soybean and tofu, lima beans and mung beans.<br>• Small quantities lean meat-based products are consumed (pork, goat meat, chicken and duck).<br>• Averaged total food intake = 1,500 kcal/day.	(Fang et al., 2015)
Wakayama and Osaka, Japan	367 community-dwelling Japanese (0-104 years old)	• ↑ abundance in *Bacteroidetes* and *Proteobacteria* (phylum level), and ↓ *Firmicutes* (phylum level) in centenarians compared to younger adults and elderly.<br>• ↑ in microbial diversity with ageing until the centenarian stage.<br>• Certain oral bacteria (*Porphyromonas, Treponema, Fusobacterium* and *Pseudoramibacter* (genus level)), which have difficulty reaching the intestinal tract due to barriers such as gastric juice and bile acid, were enriched in elderly-associated co-abundance groups. Decline in gastrointestinal tract functionality in elderly may lead to compositional changes in gut microbiota.<br>• Dietary polyphenols such as catechins found in green tea is found to significantly ↑ *Bifidobacterium* and *Lactobacillus* (genus level).<br>• ↑ *Bifidobacterium* can also be attributed to the Japanese diet rich in fermented food products.	• Located on the coast, these cities have a high consumption of seafood in addition to the typical Japanese diet consisting of short-grain rice, seaweed, green tea, and fermented food products.<br>• Other speciality dishes or ingredients include sansho pepper, soy sauce, and salt-pickled ume plum.<br>• Some individuals consume a Buddhist-vegetarian cuisine (Shojin-ryori) made of vegetables and wild plants.	(Odamaki et al., 2016; Unno and Osakabe, 2018; Tourism Exchange Division of Wakayama Prefecture, 2020)
Kyotango and Kyoto city, Kyoto Prefecture, Japan	51 centenarians from Kyotango (recognised as a longevity village), 51 centenarians from Kyoto, (65-80 years old)	• β-diversity of faecal microbiome was significantly different between regions.<br>• With urbanisation, ↑ in *Proteobacteria* and *Bacteroides* (phylum level); *Oscillospira, Parabacteroides* and *Ruminococcus* (genus level), but ↓ abundance in *Firmicutes* (phylum level); *Roseburia* and *Coprococcus* (genus level).<br>• Liposaccharide biosynthesis proteins and pathway were markedly ↑ with urbanisation, and ↓ transporters pathway and ABC transporters.	• Diet is characterised by dishes and ingredients such as porridge with red beans, longevity bento (sticky rice, preserved mackerel, beans) and soybeans (Yudofu).<br>• A common phrase to the region is to only "eat until you are 80% full".	(Fukkoshi et al., 2015; Naito et al., 2019)

Gurye, Gokseong, Sunchang and Damyang counties, Southwestern part of South Korea	30 centenarians (95-108 years old), 17 elderly (67-79 years old), 9 adults (26-43 years old) in longevity villages compared to urbanised town	• No difference in Shannon diversity index between longevity villages and urbanised town.<br>• But more diverse phyla in centenarians faecal microbiome compared to elderly and adults such *as Verrucomicrobiota*, *Proteobacteria* and *Actinobacteria* (phylum level).<br>• ↑ proportion of *Escherichia* and abundance of *Akkermansia* and *Clostridium* (genus level), but ↓ abundance in *Faecalibacterium* and *Prevotella* (genus level).<br>• Microbial metabolic pathways (phosphatidylinositol signalling system, glycosphingolipid biosynthesis, and various N-glycan biosynthesis) were predicted to be ↑, and may be associated with immune status and gut health.	• Korean cuisine is typically characterised by a high soybean-based diet including fermented soybean paste (e.g., gochujang, doenjang and cheonggukjang) as well as short-grain rice and kimchi.<br>• Seasonal wild plants and herbs are also frequently consumed (acorns, pine, Zanthoxylum piperitum, bamboo seeds, arrowroot starch, mugwort, sedum, shepherd's purse, sowthistle, aster scaber, Hemerocallis fulva, Ligularia stenocophala, Viola mandshurica, chard, mallow, dandlion, mulberry leaves, japanese ginger, aster romena, royal fern and Scilla scilloides).<br>• Egg consumption was lower in centenarians, but other protein-rich food was similar amongst centenarians, elderly and adults.	(Lee and Moon, 2013; Bong-Soo et al., 2019)
Chandel, Senapati district of Manipur, and Imphal West district, India	30 centenarians, 60 adults	• Dominance of *Erysipelotrichaceae* (family level) in gut microbiota of Indian population.<br>• ↑ *Ruminococcaceae, Rikenellaceae* and *Porphyromonadace* (family level); *Akkermansia, Alistipes, Pyramidobacter* (genus level) and ↓ *Faecalibacterium* (genus level); *Prevotellaceae* (family level) were associated with longevity in Indian centenarians.<br>• Increase in *Ruminococcaceae* diversity may be related to higher metabolic plasticity and versality of gut microbiome.	• Rural endogamous population of Naga tribes in Chandel and Senapati districts typically consume two meals/day consisting of rice and heavy meat (mainly pork and beef), and frequent consumption of rice-based fermented beverages (e.g., rice wine).<br>• In comparison, dietary pattern of sub-urban Imphal West district is composed of fish-eating vegetarians.	(Tuikhar et al., 2019)
