# Supplementary material for: Regional Diets Targeting Gut Microbial Dynamics to Support Prolonged Healthspan
Source: Front Microbiol. 2021 Apr 29;12:659465. doi: 10.3389/fmicb.2021.659465 (PMC8116520; doi:10.3389/fmicb.2021.659465)
Supplement: Supplementary file 2 [file Table_2.pdf]

Supplementary Table 2: Summary of dietary characteristics (specific foods or complex diets) and their gut microbiome-associated trends observed with individual and/or clustered biological responses specific to geography, culture, and population.

Dietary components	Description of region/population and nutritional components	Microbiota-associated trends	References
Soy and soy-products	• Isoflavones are a major component of Asian diets (15-50 mg/day) compared to Western diets (<2 mg/day).<br>• Ability to produce equol is highly variable within and between populations.<br>• 59% equol-producers amongst vegetarians compared to 25% amongst non-vegetarians (American population).<br>• 51% equol-producers in Korean-American women compared to 36% in non-Korean-American women.	• Equol, a microbial metabolite of daidzein, is produced by some strains of *Adlercreutzia, Eggerthella, Slackia, Pediococcus, Lactococcus, Bifidobacterium,* and *Clostridum* (genus level) through reduction, de-/methylation, di-/hydroxylation, and C-ring cleavage reactions.<br>• Most isolated equol-producing microbes belong to *Coriobacteriaceae* (family level), where members of this family are involved in catabolism of cholesterol-derived compounds, hinting at their functional specialisation in the gut.<br>• Presence of isoflavones in diet does not select for equol-producing bacteria.<br>• Genes involved in equol production are present in both equol- and non-equol producers.<br>• In non-equol producers, daidzein is converted into *O*-desmethylangolensin (*O*-DMA).	(Rafii, 2015; Meyer and Bennett, 2016; Vázquez et al., 2017; Mayo et al., 2019)
Wholegrain cereals (wheat, rye, oat and barley) including complex carbohydrates and resistant starch	• Wholegrain wheat and rye products are commonly consumed in German, Nordic, Russian and Australian populations, in contrast to highly refined grain products typically sold and consumed in Asian countries.<br>• Lignans, enzoxazinoids and alkylresorcinols are found in wholegrain wheat and rye products.<br>• Odd-numbered plasma alkylresorcinols are established as food-intake biomarkers that reflect medium- to long-term wholegrain consumption in populations with stable and frequent consumption.<br>• Avenanthramides (AVAs) and steroidal saponins are uniquely found in oats.<br>• (Soluble) fibres such as β-glucan in oats and arabinoxylan in wheat have been demonstrated to lower blood cholesterol and triglycerides levels as well as bile acid reabsorption.<br>• Dietary fibre content and food structure of ingested wholegrain products can influence digestibility, transit time along gastrointestinal tract, gut microbial fermentability and SCFA production in large intestine.<br>• Dietary fibres including resistant starch are recognised to play a role as prebiotics to nourish a diverse gut microbial community.	• Alkylresorcinols are metabolised to bioactive gut metabolites:<br>- 3,5-dihydroxybenzoic acid (DHBA),<br>- DHBA-glycine,<br>- 3,5-dihydroxyphenylpropionic acid (DHPPA),<br>- 5-(3,5-dihydroxyphenyl)pentanoic acid,<br>- 3,5-dihydroxycinnamic acid (DHCA).<br>• 2,4-dihydroxy-1,4-benzoxazin-3-one (DIBOA) is a main benzoxazinoid metabolite.<br>• Lignans (e.g., secoisolariciresinol) are metabolised by gut microbes into:<br>- eneterolignans,<br>- enterodiol,<br>- enterolactone<br>- by *Eggerthella* and *Peptostreptococcus* (genus level) through benzyl ether reduction, guaiacol demethylation, catechol, dihydroxylation and diol lactionisation reactions.<br>• A consortium of bacteria is required to metabolise secoisolariciresinol diglycoside to enterolactone by *Bacteroides, Clostridium, Butyribacterium, Methylotrophicum, Eubacterium, Blautia, Eggerthella, Factonifactor,* and *Longoviformis* (genus level).<br>• Variability in plasma enterolactone level is reported to be affected by gut microbial composition, lignan intake, sex, age, BMI, and ethnicity.	(Gunness et al., 2016; Hålldin et al., 2019; Landberg et al., 2019; Sanders et al., 2019; Cortés-Martín et al., 2020)

		• Vanillic acid, hydroxyhippuric acids, sulfated-conjugates of benzoic and ferulic acids as well as gut-microbial derived metabolites (DH-AVAs) have been characterised after consumption of oats.<br>• *Ruminococcus* (genus level) is a keystone species for degradation of resistant starch.<br>• Acetate and lactate are main metabolic end products of *Bifidobacteria* and lactic acid bacteria, and can be utilised by other microbes to produce propionate and butyrate (ecological network).	
Ellagitannin-containing fruits and nuts (e.g., pomegranate, berries, grapes, walnuts, almonds, and hazelnuts)	• Pomegranate, berries and nuts are seasonally available in Asian and European countries.<br>• High inter-individual variability in gut/faecal microbial composition to produce urolithins.<br>• Urolithin metabotypes have been reported to predict human host responsiveness to a polyphenol-rich intervention.<br>• Supplementation of 3,8-dihydroxy-urolithin (urolithin A) was reported to induce a molecular signature of improved mitochondrial and cellular heath.	• Ellagitannins, punicalagin and ellagic acid are metabolised to (hydroxy, dihydroxy and tetrahydroxy) urolithins by *Blautia*, *Bifidobacterium, Gordonibacter, Ellagibacter, Coprocossus,* and *Collinsella* (genus level) through lactone-ring cleavage, decarboxylation, and dihydroxylation reactions.<br>• Individuals can be stratified into three metabotypes: UM-A (25-80%), UM-B (10-50%), or UM-0 (5-25%) in various Spanish sub-populations.<br>• Metabotype is dependent on age but not affected by health status, demographics, and amount/type of ellagitannins consumed.	(Tulipani et al., 2012; Espín et al., 2013; García-Mantrana et al., 2019)
Spices (e.g., chilli, curry, curcumin, turmeric) and extracts	• Chili and curry-based dishes are frequently consumed in Asian, Indian, Mexican, African, and Turkish diets, and less frequently in Caucasian and Western diets.<br>• Chili fruit itself is not completely digested and fermented in the gastrointestinal tract as small intact material are excreted in the faecal material, and considering the residence time (~14 hours) in the large intestine, further research could investigate the two-way interaction between chilli (and other spices) and gut bacteria.<br>• Studies on spices including curcumin have shown promotion of beneficial bacteria such as *Bifidobacterium* and *Lactobacilli* (genus level) whilst reducing pathogenic strains such as *Coriobacterales, Prevotella*, *Enterococci, and Enterobacteria (genus level), suggesting a potential role in regulating intestinal microbiota and enhancement of gastrointestinal health.*	• Capsaicin was reported to increase abundance of *Ruminococcaceae* and *Lachnospiraceae* (family level) that are associated with preventing high-fat diet induced gut barrier breakdown, and reduced lipopolysaccharide (LPS)-producing family *S24_7* that is linked to obesity.<br>• Curcumin was shown to increase abundance of C*lostridium* spp., *Bacteroides* spp., *Citrobacter* spp., *Cronobacter* spp., *Enterobacter* spp., *Enterococcus* spp., *Klebsiella* spp., *Parabacteroides* spp., and *Pseudomonas* spp. (species level) but reduce *Blautia* spp. and *Ruminococcus* spp. (species level).<br>• Curcumin is poorly bioavailable, which in turn, modulates homeostasis of gut-brain axis when metabolised by gut microbiota into:<br>- 1-(4-hydroxy-3-methoxyphenyl)-2-propanol,<br>- dihydroferulic acid,<br>- ferulic acid,<br>- bisdemetylcurcumin.<br>- demethylcurcumin,<br>- dihydrocurcumin,<br>- tetrahydrocurcumin<br>- by *scherichia fergusonii, Escherichia coli, Blautia* sp., *Bifidobacteria longum*, *Bifidobacteria pseudocatenulaum*, *Enterococcus faecalis*, *Lactobacillus acidophilus*, and *Lactobacillus casei* (species level) through reduction, demethylation, acetylation and hydroxylation	(Kang et al., 2017; Shen et al., 2017; Di Meo et al., 2019; Pluta et al., 2020; Low et al., 2021)

		- reactions, and NADPH-dependent curcumin/dihydrocurcumin reductase (CurA).<br>- Black pepper, cayenne pepper, cinnamon, ginger, oregano, and rosemary were reported to enhance growth of *Bifidobacterium spp.* and *Lactobacillus spp.* (species level) but exhibit inhibitory activity against selected *Ruminococcus*, *Fusobacterium* and *Clostridium* (genus level).	
Traditional Chinese Medicine (TCM) (typically include herbs, seeds, roots, leaves, rhizomes, bark and flowers)	• As plant components, TCM or herbal medicines represent a complex mixture of phytonutrients, and have a long history in being used to alleviate symptoms, improve health and maintain homeostasis (e.g., immunity, gastrointestinal tract, brain function, eyesight) but their mechanisms are not clearly understood and validated.<br>• TCM has also been suggested to aleviate chronic liver diseases, obesity and Type ll Diabetes (T2D) via reported actions of modulating gut microbiota.<br>• Saponins in *Polygonatum kingianum* (Solomon's seal) was reported to prevent the increase in fasting blood glucose levels and alleviate T2D.	• Berberine, an isoquinoline alkaloid in *Coptis chinensis* (Chinese goldthread plant) was associated with increased abundance of *Bacteroidetes, Bifidobacterium* and *Lactobacillus* (genus level).<br>• Qushi Huayu Fang (mixture of 5 herbs: *Artemisia capillaries Thunb*, *Gardenia jasminoides Ellis*, *Fallopia Japonica*, *Curcuma longa L.* and *Hypericum Japoncium Thunb*) was associated with increased *Collinsella* (genus level) and SCFA production.<br>• Extracts of red ginseng and *Semen Coicis* (Coix seed) was shown to enhance *in vitro* growth of known probiotics, *Bifidobacterium* and *Lactobacillus* (genus level).<br>• Extract of red ginseng was also shown to inhibit growth of pathogenic strains and alleviate inflammatory bowel disease symptoms.	(Deng et al., 2012; Lu et al., 2019)
Stilbenes-containing beverages (e.g., red wine, grape juice)	• Red wine is more commonly consumed in European and Western diets, and less in Asia.<br>• Drinking 1-2 glasses of red wine/week was shown to increase gut microbial α-diversity in British women, Flemish and American populations.<br>• Gut microbiota associated with red wine consumption has been linked to lower levels of HDL cholesterol.	• Stilbenes such as piceid and resveratrol are metabolised to gut-derived metabolites:<br>- dihydro-resveratrol,<br>- lunularin,<br>- 3,4'-dihydroxy-trans-stilbene<br>- by *Coriobacteriaceae* (family level); *Bifidobacterium infantis, Lactobacillus acidophilus, Adlercreutzia equolifaciens, Slackia equolifaciens* (species level).<br>- Resveratrol can be re-glycosylated in the gut to produce piceid.	(Le Roy et al., 2020)
